# Structural Properties of Liquid SiC during Rapid Solidification

**DOI:** 10.1155/2013/273023

**Published:** 2013-10-29

**Authors:** WanJun Yan, TingHong Gao, XiaoTian Guo, YunXiang Qin, Quan Xie

**Affiliations:** ^1^Institute of New Type Optoelectronic Materials and Technology, School of Electronic Information, Guizhou University, Guiyang 550025, China; ^2^College of Electronic and Information Engineering, Anshun University, Anshun 561000, China

## Abstract

The rapid solidification of liquid silicon carbide (SiC) is studied by molecular dynamic simulation using the Tersoff potential. The structural properties of liquid and amorphous SiC are analyzed by the radial distribution function, angular distribution function, coordination number, and visualization technology. Results show that both heteronuclear and homonuclear bonds exist and no atomic segregation occurs during solidification. The bond angles of silicon and carbon atoms are distributed at around 109° and 120°, respectively, and the average coordination number is <4. Threefold carbon atoms and fourfold silicon atoms are linked together by six typical structures and ultimately form a random network of amorphous structure. The simulated results help understand the structural properties of liquid and amorphous SiC, as well as other similar semiconductor alloys.

## 1. Introduction

Interest in the properties of disordered materials is continuously growing because of their technological applications and the fundamental questions related to them. Researching the transition process of liquid to solid and the microstructure evolution law during rapid solidification are research hotspots in condensed matter physics and material science [1]. The atomic arrangement and formation processes of microstructure cannot be directly observed during rapid solidification, but detailed structural information at the atomic scale can be obtained by molecular dynamics (MD) simulations. MD simulation is widely used to study the crystallization or amorphization of liquid metals and alloys [2–5]. By contrast, studies on semiconductor alloys are limited.

Silicon carbide (SiC) is a wide-band-gap semiconductor with excellent chemical stability, electronic properties, high rigidity, and high hardness [6]. Considering that the macroscopic properties mainly depend on the SiC microstructure, a clear picture of atom packing during formation processes is important. MD simulation has been successfully used to research the mechanical properties [7], surface [8, 9], defect [10], crystal growth [11], and nanotubes or nanowires [12–15] of SiC. No theoretical study has been conducted on the structural properties of liquid SiC during solidification.

In this paper, we simulate solidification of 3C–SiC by MD using the Tersoff potential. The structural properties are analyzed in detail by the radial distribution function (RDF), angular distribution function (ADF), coordination number (CN), and visualization technology. This work is expected to present images of the structural properties of liquid SiC (*l*-SiC) and amorphous SiC (*a*-SiC) during rapid solidification, thereby providing insight into other similar semiconductor alloys.

## 2. Simulation Details and Analysis Methods

In the simulation study, 4000 Si atoms and 4000 C atoms are initially placed on the diamond lattice sites in a cubic MD cell with periodic boundary conditions. The size of the MD cell is 10*a*_0_ × 10*a*_0_ × 10*a*_0_, where *a*_0_ is the equilibrium lattice parameter of SiC (*a*_0_ = 4.43 Å). The Newtonian equations of motion are integrated using the velocity Verlet algorithm [16] with time step Δ*t* = 1 fs (1 × 10^−15^ s). The atomic interactions are described using the Tersoff potential [17], which is known to simulate the covalent system with its complex structure and energy. The Tersoff potential is also widely used for various applications of silicon, carbon, germanium, and their compounds.

To generate the liquid configuration, the SiC crystal is heated from 100 K to 4500 K, with isothermal running for 40 ps at 4500 K to achieve an equilibrium liquid state. Then, the temperature gradually decreases to 100 K at a cooling rate of 1.0 × 10^10^ K/s. The configurations are recorded every 100 K during solidification. The structural properties of *l*-SiC and *a*-SiC are analyzed by RDF, ADF, CN, and visualization technology.

## 3. Results and Discussion

### 3.1. Radial Distribution Function (RDF)

The RDF is an important method of describing the structural ordering of condensed matter systems. The Fourier transform of RDF determines the corresponding partial static structure factor obtained by X-ray imaging; thus, the RDF is an important structural parameter for comparing experimental and theoretical results.

**Figure 1:**
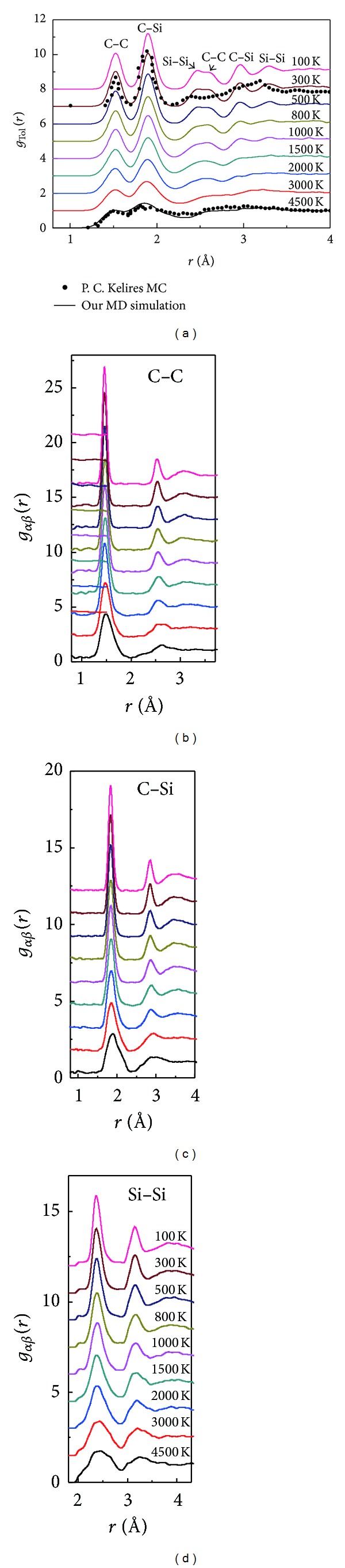
The evolution of the RDF with decreased temperature. ((a) *g*_Tol_(*r*); (b) *g*_C–C_(*r*); (c) *g*_C–Si_(*r*); (d) *g*_Si–Si_(*r*)).

Figure 1 shows the evolution of the RDF with temperature decrease. Figure 1 shows that both heteronuclear and homonuclear bonds exist in the system.  Figure 1(a) compares the results with those obtained by Kelires [18] using the Monte Carlo method (black solid round dot) at 4500 and 300 K. The first and second peaks agree well at high and low temperatures, whereas a small difference can be seen from 2.4 Å to 3.2 Å. Figures 1(b), 1(c), and 1(d) show that with decreased temperature, the first peaks of C–C, C–Si, and Si–Si all become heightened and sharp, which indicates the uniformity of the bond length and the increase in short-range order (SRO) for all kinds of bonds. The bond length of the C–C bond gradually decreases from 1.5 Å to 1.45 Å with decreased temperature from 4500 K to 100 K, and the bond length is close to that of graphite (1.46 Å). At 100 K, the second peak of *g*_C–C_(*r*) is located at ~2.55 Å, which corresponds to the distance of the C–C bond in diamond. In other words, some C atoms form C–C–C SRO during solidification. The bond length of C–Si remains 1.85 Å throughout solidification, which is very close to the results of Kaloyeros et al. [19] and Pascarelli et al. [20] as measured by extended X-ray absorption fine structure. The bond length of the Si–Si bond gradually decreases from 2.5 Å to 2.35 Å with temperature decrease. At 100 K, the bond length of Si–Si is close to that of the crystal Si at 2.36 Å, which indicates that some Si atoms form Si–Si–Si SRO.

### 3.2. Coordination Number (CN)

**Figure 2:**
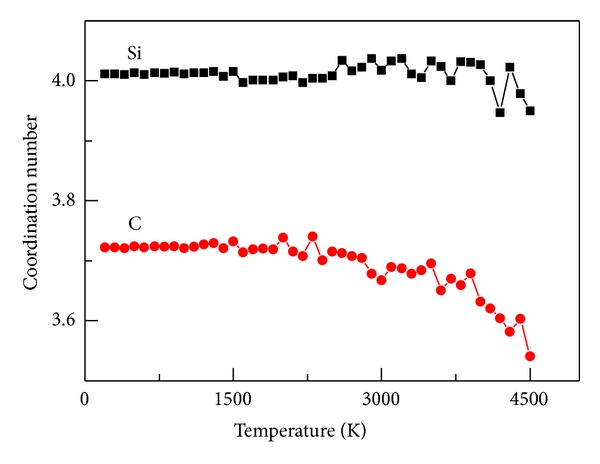
The average CN of Si and C with decreased temperature.

The CN is the average number of nearest-neighboring atoms used to describe the degree of tightness of atomic arrangement. For a multicomponent system, such a number must be separately defined for each type of atom pair. Thus, the partial coordination is indicated by the areas under the first peak (up to the minimum) in the partial RDF (PRDF) *g*_*αβ*_(*r*).  Figure 2 shows the average CN of C and Si atoms versus the temperature.

Figure 2 shows that the average CN of carbon or silicon atoms is <4 at 4500 K. The CN of Si and C increases with decreased temperature. At <1500 K, the CNs of Si and C are stable at 4.01 and 3.72, respectively. The change in the CN of silicon and carbon atoms during solidification is small, and the average CN of  SiC is 3.87 at the end of solidification, which is close to the experiment results 3.99 [20] and 3.94 [21]. In liquid SiC, neither the high-density phase of silicon (the CNs are 5.8–6.0 for pure liquid silicon [19] and 6.0 [18] for silicon in liquid SiGe) nor the liquid-liquid phase transformation occurs during liquid silicon solidification [21]. The CN of pure liquid carbon is 2.8-2.9 [19], which is lower than that of SiC.

Table 1 shows the positions of the first three peaks of the RDF and the average of corresponding partial CNs at 100 K.

In Table 1, 1.68 silicon and 2.33 carbon atoms on average are bonded to each silicon atom, that is, *z*_Si–Si_ = 1.68 and *z*_Si–C_ = 2.33, whereas 1.39 carbon and 2.33 silicon atoms are bonded to each carbon atom, that is, *z*_C–C_ = 1.39 and *z*_C–Si_ = 2.33. In total, silicon has 4.01 nearest neighbors (*z*_Si_ = 4.01) and carbon has 3.72 nearest neighbors (*z*_C_ = 3.72). The final structure of quenched SiC cannot be described as a tetrahedral network because most carbon atoms are threefold coordinated.

### 3.3. Angular Distribution Function (ADF)

**Figure 3:**
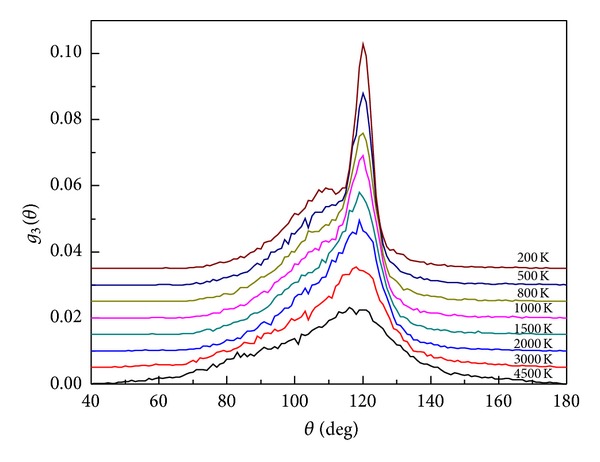
The evolution of the ADF with decreased temperature.

**Figure 4:**
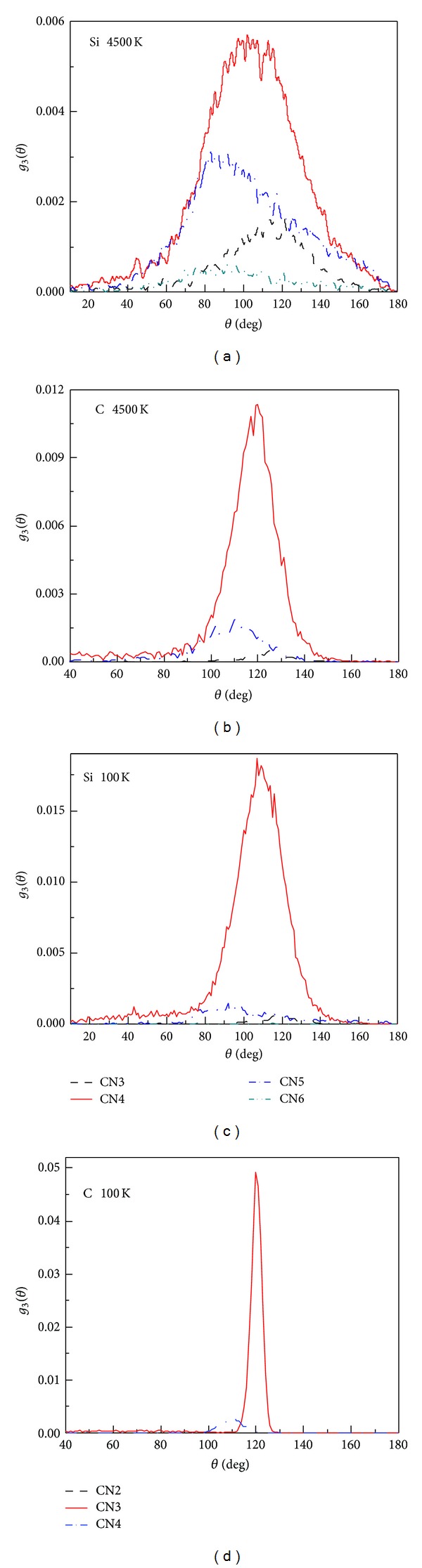
The ADF of SiC at 4500 K and 100 K. ((a), (c) at 4500 K and 100 K, Si as central atom, CN-3, 4, 5, 6; (b), (d) at 4500 K and  100 K, C as central atom, CN-2, 3, 4).

The ADF can be used to describe the statistical average of angles formed with neighboring atoms.  Figure 3 shows the relationship between the ADF and temperature.  Figure 3 shows that the maximum peak of the ADF is located at 120°, which indicates that many threefold coordinated atoms exist in the system. Angular distribution near 109° indicates some nonstandard tetrahedron units or other complex local structures. With decreased temperature, the range of the ADF narrows and the maximum peak gradually increases, which indicates that the atomic structure becomes more orderly. To reveal the transformation of the ADF, the ADF of silicon and carbon as center atoms are analyzed in detail.  Figure 4 shows the ADF of silicon and carbon as center atoms at 4500 and 100 K.

Figures 4(a) and 4(b) show that at 4500 K, complex bond types exist in liquid SiC. Silicon atoms mainly form threefold, fourfold, fivefold, and sixfold coordination structures, and fourfold silicon atoms account for the greatest proportion. The bond angles of fourfold, fivefold, and threefold silicon atoms are distributed within the ranges of 96°–109°, ~90°, and 92°–128°, respectively. Carbon atoms mainly form threefold, fourfold, and a small number of twofold coordination structures. The bond angles of threefold and fourfold carbon atoms are distributed within the ranges of 115°–121° and 97°–121°, respectively. Figures 4(c) and 4(d) show that silicon atoms mainly form fourfold coordination structures at 100 K, and the main peak is located at ~109° (bond angle of tetrahedral). Carbon atoms mainly form a large number of threefold coordination structures, and the main peak is located at ~120° (bond angle of graphite). During solidification, silicon atoms tend to form fourfold coordination structures with a tetrahedral angle, whereas carbon atoms tend to form threefold coordination structures with a graphite angle. However, no graphite structure forms at the end of solidification, as discussed in the following section.

### 3.4. Microstructure Visualization Analysis

**Figure 5:**
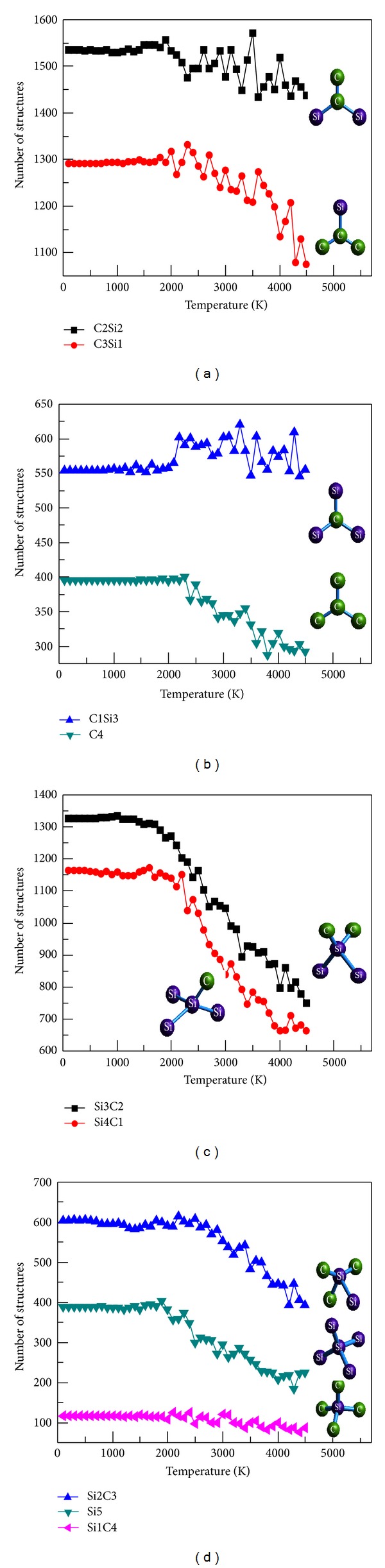
The number of main structures of C or Si as central atom with temperature decrease. ((a), (b) threefold coordination structures of C as central atom; (c), (d) fourfold coordination structures of Si as central atom).

To present a clear image of microstructures during solidification, Figure 5 shows a change in the main structures of silicon and carbon as the center atoms with decreased temperature. We use A*m*B*n* to denote the structure. A is the center atom type and *m* is the number of A. Meanwhile, B is the atom type that differs from A and *n* is the number of B. For example, C3Si1 represents the carbon as the center atom, and the structure comprises three carbon atoms and one silicon atom. The green ball represents carbon atom, and the purple one represents silicon atom.

When carbon is the center atom, four kinds of threefold coordination structures exist, namely, C1Si3, C2Si2, C3Si1, and C4. Figures 5(a) and 5(b) show that the number of the four kinds of structures from more to less is C2Si2, C3Si1, C1Si3, and C4. C4 has the least number of structures that indicates the absence of carbon atomic segregation throughout the entire solidification process. The number of the four kinds of structures during oscillation increases with decreased temperature from 4500 K to 1500 K and gradually tends to be stable when the temperature is <1500 K. When silicon is the center atom, five kinds of fourfold coordination structures exist: Si1C4, Si2C3, Si3C2, Si4C1, and Si5. Figures 5(c) and 5(d) show that the number of the five kinds of structures from more to less is Si3C2, Si4C1, Si2C3, Si5, and Si1C4. Very few Si5 and Si1C4 structures exist, indicating that no silicon atomic segregation occurs and no crystal structure forms throughout the entire solidification process.

**Figure 6:**
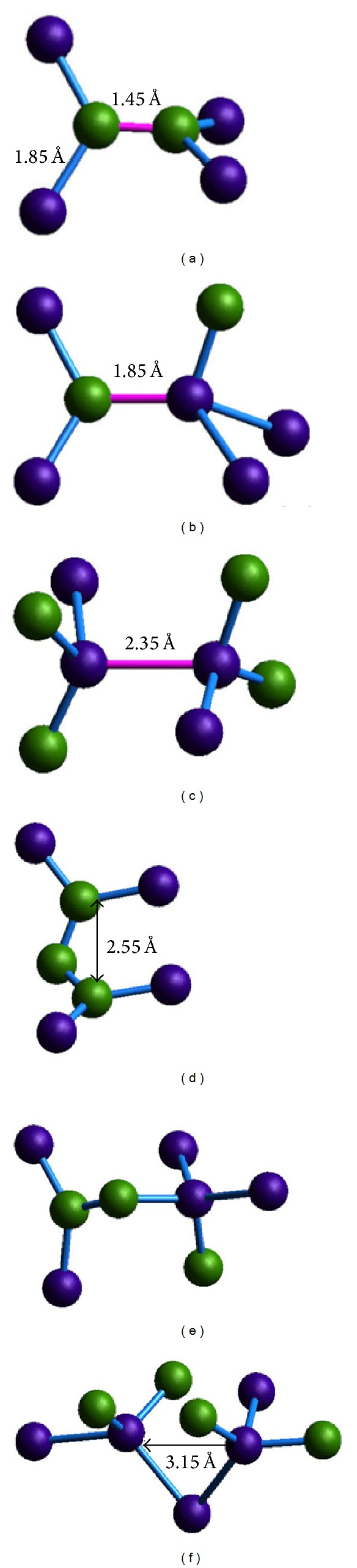
Six typical combination modes of carbon atoms and silicon atoms. ((a), (b), and (c) central atom is the neighbor atom of 3-3, 3-4, 4-4 coordination, (d), (e), and (f) sharing neighbor atom of 3-3, 3-4, 4-4 coordination).

In consideration of the threefold carbon atoms and fourfold silicon atoms accounting for a large proportion after solidification, the combination mode for these atoms must be intensively investigated.  Figure 6 shows the six typical combination modes of threefold carbon atoms and fourfold silicon atoms.

Figures 6(a), 6(b), and 6(c) represent the central atom as the neighboring atom of the other structure (the red bond connects two center atoms).  Figure 6(a) shows two threefold coordination structures connected by two carbon atoms that are the center atoms of the two structures, and the two structures are not in the same plane. The bond angles of Si–C–Si and C–C–Si are both ~120°, and the lengths of the C–C and C–Si bonds are ~1.45 and ~1.85 Å, respectively, which corresponds to the location of the first peaks of *g*_C–C_(*r*) and *g*_C–Si_(*r*) in Figures 1(b) and 1(c).  Figure 6(b) shows a threefold and a fourfold coordination structure connected by a carbon atom and a silicon atom, which are the center atoms of the two structures.  Figure 6(c) shows two fourfold coordination structures connected by two silicon atoms, which are the center atoms of the two structures. Figures 6(d), 6(e), and 6(f) show the connection of the two structures by sharing a neighboring atom.  Figure 6(d) shows two threefold coordination structures connected by sharing a neighbor carbon atom and forming a short-range structure C–C–C. The distance of the two carbon atoms farthermost apart is ~2.55 Å, which corresponds to the location of the second peak of *g*_C–C_(*r*) in Figure 1(b). Two structures form a twisty chain structure.  Figure 6(e) shows a threefold and a fourfold coordination structure connected by sharing the neighboring carbon atom.  Figure 6(f) shows two fourfold coordination structures connected by sharing the neighboring silicon atom and forming a short-range structure Si–Si–Si. The distance of the two farthermost silicon atoms is ~3.15 Å, which corresponds to the location of the second peak of *g*_Si–Si_(*r*) in Figure 1(c).

**Figure 7:**
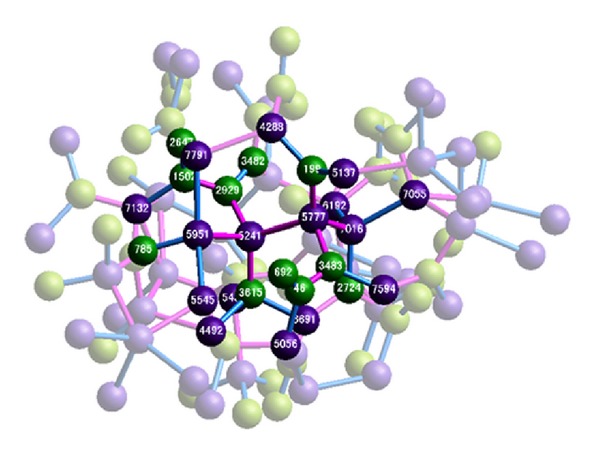
LAN amorphous structure at the end of solidification of SiC.

Figure 7 is the local area network-like amorphous structure at the end of the solidification of liquid SiC, and the six typical structures above-mentioned are illustrated in Figure 7. Threefold carbon atoms are not the unsaturated bond or dangling bonds of a tetrahedral mesh but form a graphite-like structure (bond angle, 120°; bond length, ~1.46 Å). Carbon atoms with numbers. 2647, 1502, 2929, and 3482 form a C–C–C–C short-range structure. Ultimately, no hexagonal graphite structure forms in the end because of the distorted connection. Five center silicon atoms with numbers. 5951, 5241, 5777, 7016, and 7055 of fourfold coordination structures connected individually by each center atom to form a diamond-like structure (bond angle, 109°; bond length, ~2.35 Å). No diamond structure forms in the end because the coordination atoms of silicon deviate from the crystal lattice positions of SiC. A random network amorphous structure is formed by connecting the threefold carbon atoms and fourfold silicon atoms at the end of solidification.

## 4. Conclusions

The rapid solidification of liquid SiC containing 8000 atoms is simulated with the Tersoff potential using MD. The microstructure properties of liquid and amorphous SiC are analyzed in detail by RDF, ADF, CN, and visualization technology. Results show that with decreased temperature, the distance of C–C and Si–Si bonds gradually decreases, the C–Si bond remains at 1.85 Å, and the SRO increases during solidification. Heteronuclear and homonuclear bonds coexist and no carbon or silicon atomic segregation occurs in the system. The average CN of SiC is <4 throughout the entire solidification process. Silicon atoms mainly form fourfold coordination structures with a tetrahedron bond angle of 109°, and carbon atoms mainly form threefold coordination-based structure with a graphite bond angle of 120° during solidification. The short-range structure is distorted because of the C–C–C connection. The coordination atoms of silicon deviate from the crystal lattice positions of SiC. Ultimately, no hexagonal ring of graphite and diamond structure forms. A random network amorphous structure is formed by connecting threefold coordination structures of C and fourfold coordination structures of Si at the end of solidification.

## Figures and Tables

**Table 1:** Position of the first three peaks of RDF and the average of corresponding partial CN *z* at 100 K.

	*r*_1_/Å	*r*_2_/Å	*r*_3_/Å	*z*
C–C	1.45 (1.46)^a^	2.55 (2.52)^b^	3.05 (3.07)^c^	1.39 (1.19)^e^
C–Si	1.85 (1.88)^c^	2.85	3.40 (3.60)^c^	2.33 (2.27)^e^
Si–Si	2.35 (2.36)^d^	3.15 (3.07)^c^	3.90 (3.84)^d^	1.68 (1.75)^e^

^a^Graphite, ^b^diamond, ^c^SiC crystal, ^d^Si crystal, and ^e^Kelires [18].
